# How odor cues help to optimize learning during sleep in a real life-setting

**DOI:** 10.1038/s41598-020-57613-7

**Published:** 2020-01-27

**Authors:** Franziska Neumann, Vitus Oberhauser, Jürgen Kornmeier

**Affiliations:** 1 Institute for Frontier Areas of Psychology and Mental Health, Freiburg, Germany; 2 Faculty of Biology, University of Freiburg, Freiburg, Germany; 3 Department of Psychiatry and Psychotherapy, Medical Center, University of Freiburg, Freiburg, Germany; 4 Faculty of Medicine, University of Freiburg, Freiburg, Germany

**Keywords:** Consolidation, Long-term memory

## Abstract

Effortless learning during sleep is everybody’s dream. Several studies found that presenting odor cues during learning and selectively during slow wave sleep increases learning success. The current study extends previous research in three aspects to test for optimization and practical applicability of this cueing effect: We (1) performed a field study of vocabulary-learning in a regular school setting, (2) stimulated with odor cues during the whole night without sleep monitoring, and (3) applied the odor additionally as retrieval cue in a subsequent test. We found an odor cueing effect with comparable effect sizes (d between 0.6 and 1.2) as studies with sleep monitoring and selective cueing. Further, we observed some (non-significant) indication for a further performance benefit with additional cueing during the recall test. Our results replicate previous findings and provide important extensions: First, the odor effect also works outside the lab. Second, continuous cueing at night produces similar effect sizes as a study with selective cueing in specific sleep stages. Whether odor cueing during memory recall further increases memory performance hast to be shown in future studies. Overall, our results extend the knowledge on odor cueing effects and provide a realistic practical perspective on it.

## Introduction

Our memories allow us to know what to do within the next hour, the next day or even the next year; in other words, to make plans about the future based on memorized past experiences. Memory allows us to understand language, thus to communicate with people, to draw causal relations and to adequately disambiguate and interpret the incomplete and noisy information available to our senses^1–4^. Further, (episodic) memories of our personal experiences strongly influence our personalities (e.g.^5^). We transfer 1 to 10 Mbit of information per second from our eyes to the primary visual cortex (e.g.^6^). Similar magnitudes of information may be transferred within the other sensory modalities. However, only a fraction of this information enters consciousness. And only a fraction of the conscious (but also partly unconscious) information in turn enters long-term memory. Empirical and in particular patient studies teach us about short-term and long-term memory systems^7^. Patient cases, such as the one of Henry Molaison, impressively demonstrate the role of the hippocampus for short-term memory storage and the integration into long-term memory^8,9^. We have learned that parts of the short-term memorized information are reactivated in the hippocampus during slow-wave sleep periods at night in order to become redistributed within preexisting networks including the neocortex^10–13^.

The critical question for each of us and for the human society as a whole is, how the brain decides what to memorize and what to forget, and how we can influence this decision most efficiently. Emotional labels can be influential^14^. Also, what repeatedly occurs is worth being remembered (e.g.^15,16^).

One elegant way to actively influence, which selective information will be kept in long-term memory, has been described by Rasch *et al*. in 2007^17^. They found that presenting odor cues during learning of object locations in a 2D object-location task and re-presenting the same odor cues during slow wave sleep (SWS) improves memory consolidation. The current explanation is that during SWS a subset of the content in the hippocampal short-term memory is reactivated and thereby integrated into preexisting networks located in the neocortex. Re-presenting the odor cues during SWS triggers the selection of content associated with the odor cue, as the odor had been presented during the previous encoding period, thus increasing the probability of the content being integrated into long-term memory. Interestingly, this cueing seems to work independently of whether the odor cue was experienced as pleasant or unpleasant^18^.

Rasch *et al*.’s findings and several subsequent studies arising from their seminal work impressively demonstrated how specific cues during learning and during sleep can influence which content will be transferred from short-term to long-term memory (targeted memory reactivation, “TMR”, for recent reviews see^13,19,20^). Accordingly, these findings are very important from both theoretical and practical points of view. However, at the same time, they are restricted to highly controlled laboratory settings (see^21^ for an exception where participants slept at home). Practical relevance of the TMR for real life learning situations, as for example in school settings, is unclear so far. One possible problem for a potential transfer of the TMR to practical applications is the timing of the odor cues during sleep. Memory and sleep research have shown that SWS is the critical sleep stage for memory consolidation (e.g.^22^). Accordingly, most studies monitored sleep stages and applied the odor cues exactly during the SWS (or other stages) of their participants’ sleep. The necessities of sleep monitoring with EEG and timely odor applications may be considerable obstacles for easy and inexpensive real-life applications of the basic cueing principles.

Given this background, the present study had three aims: (1) Does the basic principle of cueing learning content during the encoding period and during sleep for selective memory consolidation also work in real life situations? (2) Does an unselective odor cue application during the whole night, and thus not only during selected SWS stages, also work? (3) Do memory cues, presented during encoding and during the consolidation at night, further improve memory if they are again presented during memory retrieval?

Positive answers to these research questions would push this basic principle of memory cueing towards an easy and inexpensive practical application.

Accordingly, our study was set out to examine the effect of rose odor cues on the memory performance of eleven and twelve-years-old German students, given in different learning contexts, as well as during the students’ sleep at home, and during memory retrieval.

## Methods

### Participants

The study was conducted with two 6th grade classes consisting of 32 (18 girls and 14 boys) and 22 (12 girls and 10 boys) healthy eleven- and twelve-years old German students in their normal school and home environments and contained no invasive measurement. The students had one year of English classes prior to the study. Written informed consent was obtained from all parents beforehand. The study was in accordance with the Declaration of Helsinki as confirmed by the local ethics committee.

### Experimental paradigm

In order to analyse the effect of odor cues in a school setting, we used vocabulary tests as academic examination tools, as they are performed regularly in second language classrooms. We applied a mixed design with four within-group conditions and two experimental groups. 54 participants from two different school classes (Class 1 with 32 students and Class 2 with 22 students) took part in this study. For each class, half of the students were randomly assigned to the test and control groups respectively. Accordingly, the test group contained 15 girls (9 from Class 1 and 6 from Class 2) and 12 boys (7 from Class 1 and 5 from Class 2). The control group contained 15 girls (9 from Class 1 and 6 from Class 2) and 12 boys (7 from Class 1 and 5 from Class 2). All students learned and were tested in four separate sets of German-English vocabulary pairs over the course of the experiment. The respective vocabulary sets were introduced to all students during the school lessons without any odor cue. Similar to the study by Rasch et al^17^, we used rose fragrance as odor cue. The odor cue was first applied to the test group while they were studying the English vocabulary at home.

In *condition “N”* none of the participants received any odor cue. In the *condition “LT”* the test participants were exposed to rose fragrance as odor cue during the vocabulary learning periods at home and during the vocabulary test at school, which took place one week after the initial presentation of the vocabulary unit by the teacher.

In the *condition “LS”* participants received the odor cue during the learning period at home as well as every night of the week before the test. At night, the odor cue was present during the whole duration of the sleeping period.

In the *condition “LST”* participants received the odor cue during the learning period at home, every night while sleeping at home and during the subsequent vocabulary test at school, seven days after the learning unit.

**Figure 1:**
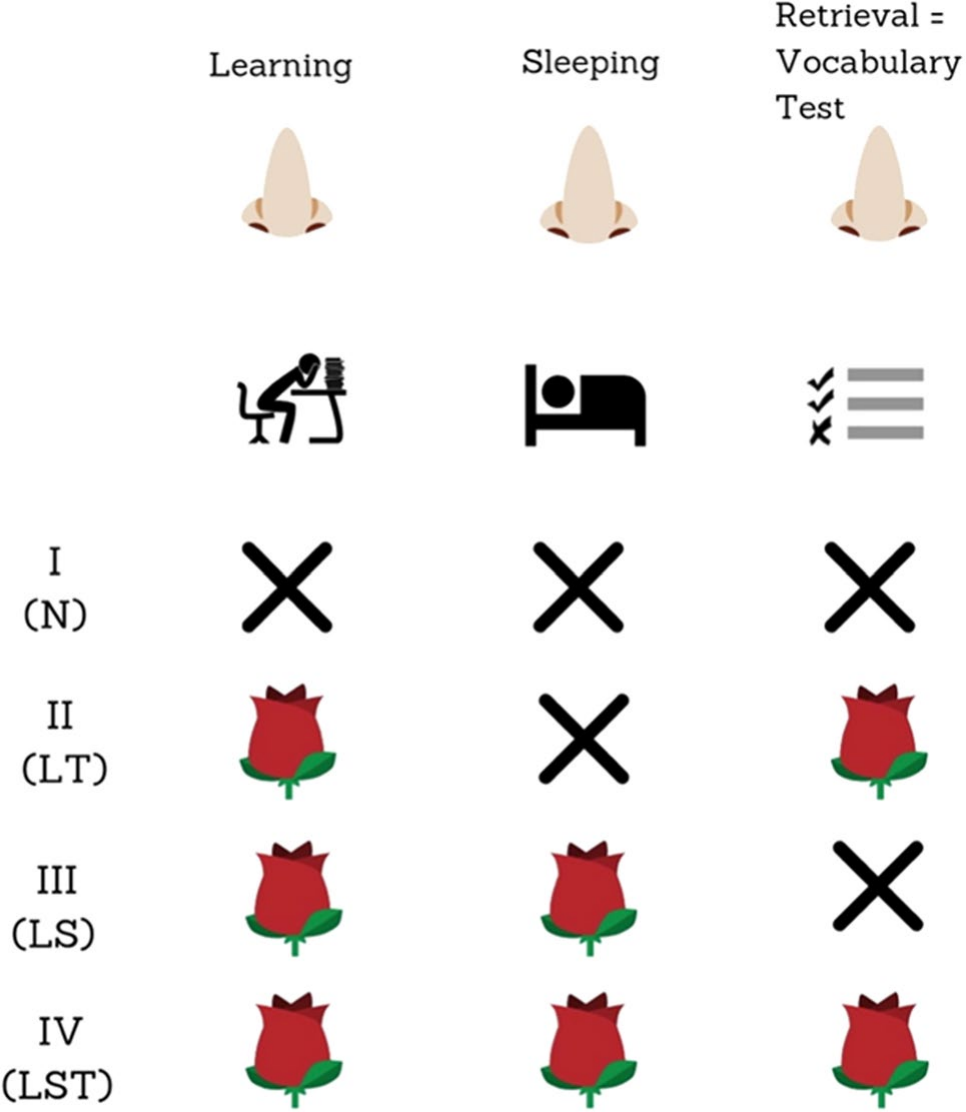
Paradigm: The study consisted of four steps: (I) Initial presentation of the material at school (II) Learning at home, (III) Sleep at home (7 nights) and (IV) a vocabulary test at school 7 days after the learning unit. No odor cue was applied in Condition N. In the LT condition, students from the test group received odor cues during learning at home (L) and during the vocabulary test (T) seven days after the learning unit at school. In condition LS, they received odor cues during learning and during sleep in seven successive nights at home. Finally, in the condition LST, they received odor cues during learning at home, during sleep at home and during the final vocabulary test at school. Students from the control group learned the identical vocabulary material as the test group, but received no odor cues neither during learning nor during sleep and during the tests.

For a graphical description of the different conditions see Fig. 1.

One constraint of the real-life setting in the present study was that the four vocabulary units, which were used for the four experimental conditions (N, LT, LS, LST), were taught successively over four weeks as parts of the predetermined school curriculum. Variable levels of difficulty between vocabulary units and therefore between conditions may thus be a potential confounder. We addressed this problem by dividing each of the two classes into a test and a control group. Both groups had learned and were tested on the same vocabulary sets, but the control group did not receive any odor cue. The between-group-comparisons are thus not affected by varying levels of difficulty between vocabulary units and tests. However, one must keep this potential confounder in mind, when regarding between-condition comparisons as well as interaction effects.

Due to some school internal reasons, the LST condition was unfortunately not executed in Class 2. As a result, only data from the N, LT and LS conditions were available for Class 2. Further, the order of the experimental conditions was fixed for all participants.

Odor cues were applied via conventional commercially available incense sticks. All sticks were used in an unlit state, as they already distributed a highly intense fragrance as such. Depending on the experimental condition, the students were instructed to put the incense stick next to them on their desk while they were studying the English vocabulary at home, on their nightstand next to their bed while they were sleeping, and/or on their desk during the vocabulary test itself, which took place seven days after the initial presentation of the vocabulary in school. Having the incense stick on the nightstand during the whole night implied that the rose fragrance was supplied during the complete sleep period and in all sleep cycles.

### Data analysis

The number of errors made in the vocabulary tests served as the measure for the learning success, with smaller numbers of errors indicating better memory performance. The vocabulary tests worked as follows: The students had to translate German words into the equivalent English translation as well as vice versa. Furthermore, the students had to form own sentences and complete given sentences with these words. The test consisted of 20 vocabulary words for Class 1 and 30 words for Class 2. An error was defined as a word (1) which was written wrongly, (2) which wasn’t translated correctly, or (3) not remembered at all, indicating a failed retrieval of memorized information.

We first normalized the data with respect to the number of vocabulary words tested in the respective vocabulary tests, resulting in error rates (i.e. percent errors). We then compared the performances between conditions and groups with a first ANOVA with the between factors CLASS (Classes 1 and 2), GROUP (test and control group with and without odor cue respectively) and the within-factor CONDITION (N, LS, LT). The condition LST did not enter this first ANOVA, because no data from Class 2 was available for this condition. A second ANOVA was applied to only the data from Class 1 with the between-factor GROUP. Based on the results from ANOVA 1 and the related post-hoc tests, we restricted ANOVA 2 to the conditions LS and LST and excluded the factor CLASS as well.

Post-hoc tests are typically calculated only, if justified by significant ANOVA results. In the present study we calculated post-hoc tests independent of the ANOVA results by the following reason: One potential confounding factor for the results of the two ANOVAs was the fact, that different vocabulary materials and therewith different final tests were used for the different conditions. This was due to the fact that this field study was integrated into the regular school curriculum and the different conditions were realized with different English teaching units. This may have introduced additional noise to the comparison between conditions, affecting the results of the ANOVA factor CONDITION, as well as the interaction between CONDITION and GROUP. In order to get a clearer picture we thus calculated additional randomization tests comparing the test and control group results for each condition separately and report p-values corrected for multiple testing according to Bonferroni-Holm^23^ in Table 1 below.

**Table 1:** Post-hoc tests corrected for multiple testing.

Conditions (test versus control)	p-values permutation test (uncorrected)	p-values permutation test (corrected)	Effect Sizes (Cohen’s d)	Remarks
N	0.3637	0.3	0.085	Data from Class 1 & 2
LT	0.2397	0.2	0.19	Data from Class 1 & 2
LS	0.0147	0.04	0.61	Data from Class 1 & 2
LS	0.0421	0.08	0.64	Data from Class 1
LST	0.0004	0.0016	1.22	Data from Class 1

Randomization tests are variants of permutation tests. The basic idea of a permutation test is, to generate reference distributions out of the measured data instead of relying on theoretical distributions. This is done by permuting the assignment of the measured data to the experimental groups. If the total number of available permutations is too large, only a random sample of permutations will be selected. In this case, the test is called “randomization test”.

We report partial eta square (*η*^2^_*p*_) and Cohen’s d as effect size estimates.

## Results

The first ANOVA (including data from both classes) revealed neither effect for the factor CLASS (p = 0.072, F(1,50) = 3.39, *η*^2^_*p*_ = 0.12) nor for the factor GROUP (p = 0.26, F(1,50) = 1.31, *η*^2^_*p*_ = 0.053) nor for CONDITION (p = 0.44, F(2,100) = 0.82, *η*^2^_*p*_ = 0.016). However the ANOVA revealed a significant GROUP x CONDITION interaction (p = 0.016, F(2,100) = 4.28 *η*^2^_*p*_ = 0.079).

The post-hoc permutation tests revealed significant differences between test and control group for the condition LS (p = 0.04, Cohen’s d = 0.61) but not for the condition LT (p = 0.24, Cohen’s d = 0.2) nor for the condition N (p = 0.4, Cohen’s d = 0.08, see Table 1 for an overview).

The second ANOVA (including only data from Class 1) revealed a significant effect for the factor GROUP (p = 0.0078, F(1,30) = 8.15, *η*^2^_*p*_ = 0.44), indicating better memory performance in the test group than in the control group. Further, the ANOVA revealed a significant effect for the factor CONDITION (p = 0.0089, F(1,30) = 7.82, *η*^2^_*p*_ = 0.21), indicating larger memory effects for condition LST compared to condition LS. For the interaction (Group x Condition) the ANOVA revealed no significant effect (p = 0.44, F(1,28) = 0.6, *η*^2^_*p*_ = 0.02). We also calculated post-hoc permutation tests between test and control group for the condition LST (only data from Class 1). The permutation test revealed a corrected p = 0.001 with d = 1.22. For an overview about post-hoc tests see Table 1.

**Figure 2:**
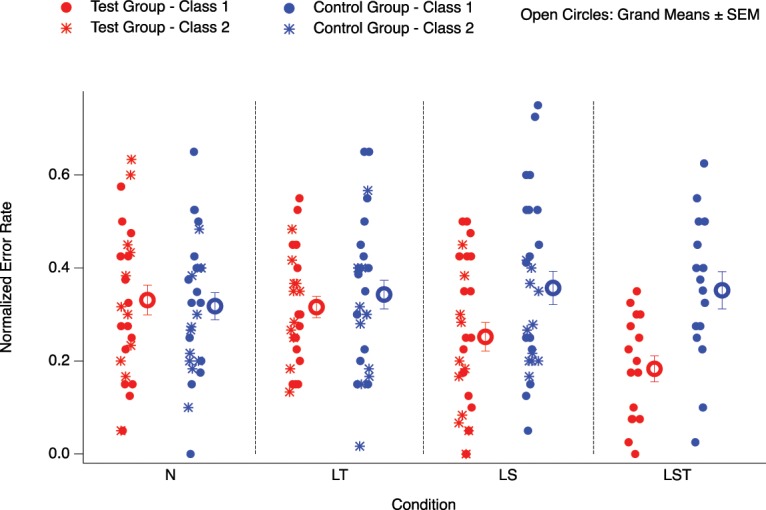
Results: Small icons (stars and circles) represent data from individual participants. The data of the test group is in red, of the control group is blue. Larger open circles represent averages across classes ± SEM. Filled circles represent data from Class 1, stars represent data from Class 2. The different columns, separated by vertical dashed black lines, represent the different experimental conditions (N: no odor cue; LT: odor cue during learning and test; LS: odor cue during learning and during sleep; LST: odor cue during learning, sleep and test). Horizontal jitter across icons within monochrome sub-columns is due to presentation purposes. We found smaller numbers of errors LS and LST compared to conditions N and LT, and a tendency for smaller errors in LST compared to LS. Class 2 provided no data for condition LST. Data are normalized with respect to the number of vocabulary words tested in the respective vocabulary tests.

Figure 2 shows both grand means (±SEM) from test (red color) and control groups (blue color) separately for each experimental condition (open circles) together with data from the individual participants. The data is normalized with respect to the number of words tested in the vocabulary tests. Data from Class 1 (filled circles) and Class 2 (stars) are averaged together, except for condition LST, where only data from Class 1 were available. Table 2 lists the normalized mean and standard deviation values separately for both groups and for the different experimental conditions.

**Table 2:** Grand Means (±SD) of the normalized error rates.

	N	LT	LS	LST
Test Group	0.33 (0.17)	0.32 (0.12)	0.25 (0.16)	0.18 (0.11)
Control Group	0.32 (0.15)	0.34 (0.16)	0.36 (0.19)	0.35 (0.16)

Notice that some of the individual data points could be regarded as outliers, (see Fig. 2 and the Supplementary Fig. 1) potentially increasing the alpha error probability. We address this outlier issue in the Supplementary Material.

## Discussion

Several studies show that short-term memory content is integrated into cortical long-term memory during slow-wave sleep and that this process can be selectively influenced by a combined application of odor cues during encoding and during slow-wave sleep at night (targeted memory reactivation, TMR). In the present study, we replicated this beneficial effect of an odor cue. In particular, we found that (1) odor cues not only work in a lab environment, but also with students in a regular school setting; that (2) odor cues also take effect if presented continuously during the complete sleep period at night, rather than selectively during slow-wave sleep stages. (3) We observed some indirect indication for a further performance benefit with additional cueing during the recall test.

### Real life settings and effect sizes

Leaving the experimental lab environments and doing field research in real life situations is a priori challenging. In a lab, most of the potentially confounding parameters can be controlled and their influence can be minimized. This is not the case in real life situations. Confounding influences typically increase the noise factors of the statistical terms and ultimately reduce the effect sizes. The following constraints need to be kept in mind, with respect to the present results:

- Due to organisational reasons, the embedding of the current study in the daily school routine did neither allow a new allocations of the students to either test and control group for each experimental condition, nor a randomization of the order of conditions between students within the two groups. It is thus possible that at least part of the reported effects may reflect an order effect.
- The vocabulary learning units as well as the vocabulary tests in school had been conducted by two different teachers in two different school classes and with slightly different vocabulary tests. Therefore, there might be small differences in the instructions given to the students and other realization details.
- We had no control over how often the students repeated the learning material at home within the seven days before the final test.
- We had to rely on the students’ reports on their use of the odor cues while studying at home and during their sleep in the seven nights between the initial encoding of the vocabulary material and the final test.
- We had no control over the spatial distance between the odor cue and the students (i.e. individual odor intensity) while they were studying at home, during sleep, and during the test itself.
- Several studies reported about reduction of sleep time and changes in sleep pattern (reduction in EEG amplitudes and a linear increase in peak spectral frequency of EEG sleep spindles) during adolescence within an age range between 11 and 17^24,25^. We did not record sleep EEG and had no control about the students’ absolute sleep time or their sleep quality. However, if a reduction of sleep duration and/or a change in sleep pattern did affect consolidation of our student participants negatively, the control group must have been affected more strongly than the test group, given the current results. Also, the test group must have been selectively more affected in the N (no odor cue) and LT (odor cues during learning and the test but not during sleep) conditions. Of course, in principle, it might be possible that the students in the test group slept longer only and selectively in the conditions containing an odor cue during sleep (LS and LST). But it is not very probable. Future (lab and field) studies may investigate the relation between adolescent changes in sleep pattern and duration and the efficacy of odor cueing during sleep.
- The different experimental conditions were based on different vocabulary material. Within-group comparisons across experimental conditions may thus be confounded by variations in the difficulty of the vocabulary material and/or final tests. Comparisons between test and control groups within conditions are unaffected by this factor. However, comparisons between conditions and interaction tests may have been affected by this.
- Time-on-test, potential verbal and non-verbal interactions between students during tests had not been perfectly controlled.

Given that all of these factors (except the first) could have introduced additional “experimental noise” into our data, it is remarkable that our effect sizes with Cohen’s d between 0.6 and 1.2 are in the same range as, or larger than the effect size (d ≈ 0.6) in Rasch *et al*.’s study^15^ (we have estimated this value from the leftmost graph of their Fig. 2A).

### Control conditions

Odor cueing paradigms typically include test conditions in which a vehicle contains an odor, which is then distributed at different steps during the learning process. These conditions are contrasted with control conditions in which the vehicle is either presented without the odor or with varying odors (e.g.^18^). Unfortunately, no vehicle had been distributed to the participants of the control conditions in the present study. This may in principle make our results vulnerable to psychological expectancy effects. However, the following argumentation makes this an unlikely explanation for our findings.

The odor cue effect is only present if the odor cue had been given during night. No difference is found between test and control groups in the LT condition (odor during learning and during test – but not during sleep). In contrast, for both the LS (odor during learning and during sleep) and LST (odor during learning, sleep and final test) conditions, we found significant differences between the test and control groups (keep in mind that the results from the LS condition is based on data from the two classes, whereas the LST condition is restricted to data from Class 1). It is very unlikely that a psychological expectancy is selectively coupled to receiving the odor device at night (LS and LST conditions), but not for receiving it during the learning period (LT condition). Further, a recent study, applying auditory word cues in the context of a vocabulary-learning paradigm, found that memory performance of those words not having been cued at night is similar to the memory performance of words in a condition without any cue during night^26^. If psychological expectancy would completely explain the effect of memory cueing during sleep, it should not be restricted to a subset of words being cued at night.

### Odor as contextual cue for learning

Odor has already been used as a cue for context-dependent memory formation prior to the findings established by Rasch and his colleagues in their seminal study^17,27,28^. Typically, odor cues had only been presented during encoding and during retrieval, but not during sleep. It was shown that “context” is not necessarily a spatial parameter, but that fragrances can also establish an olfactory context, which can improve later retrieval (e.g.^27,28^).

It is possible that the odor-context-dependent memory effects and the effects of odor cueing during learning and during sleep are based on the same underlying mechanisms. It is also possible that the impact of an odor on the retrieval of information is independent of its impact on consolidation. These earlier studies may have shown retrieval effects, and odor-during-sleep studies may have shown consolidation effects. Our study shows significant consolidation effects and some indication for beneficial retrieval with odor cues (twice as large effect sizes in condition LST than in condition LS but no significant ANOVA interaction). The question remains, why we did not see any pattern of cueing effects in the LT condition? One possible explanation is the larger inherent noise level of the present field study compared to better-controlled laboratory studies.

### Do odor cues during sleep improve both memory consolidation and memory retrieval?

The formation of memories is typically divided into three major steps: encoding, consolidation and retrieval^29^. If we are not able to recall a certain past event or fact, the relevant information may not have been consolidated or, alternatively, it has been consolidated, i.e. the information is somewhere in our memories, but we cannot retrieve it. So far, several studies have shown that cueing during learning and during SWS increases memory performance. Given the state of the art in memory research, this improvement was interpreted as an improvement of the memory consolidation processes (e.g.^19^). However, memory performance tests, conducted several days after the cueing during SWS, always test both consolidation and retrieval success. In the current study, we added cueing during retrieval as another experimental parameter in order to disentangle both effects and to test for further memory improvement.

Testing the effects of odor cues on memory retrieval was unfortunately restricted to the data sets from Class 1, as no data from Class 2 was available for the LST condition. A significant interaction between the factors GROUP (test vs. control) and CONDITION (LS vs LST) in the second ANOVA would have indicated an effect of odor cueing on memory retrieval during test. However, the ANOVA did not reveal a significant interaction. Despite the absence of a significant ANOVA interaction we calculated post-hoc tests for the conditions LS and LST, comparing test performance of test and control groups. In our view, this is reasonable because the comparison between conditions – as part of the interaction test – can be confounded by the different vocabulary materials used in the different conditions, as already discussed above. The post-hoc tests revealed significantly better memory performance of the test group compared to the control group with a considerably large effect size (d = 1.23) for the condition LST and with half the effect size for the condition LS (d = 0.6). No significant difference between groups was found for the conditions LT and N (see also Table 1 for an overview).

An effect size (d = 1.22) twice as large for condition LST than for condition LS (d = 0.6), indicates some additional influence of the odor cue for retrieval. However with regard of the results of the ANOVA this point remains controversial. Our pattern of results motivates at least a second look on combined cueing during learning, sleep *and* retrieval in a future repetition of the current study.

### One and the same odor cue for different learning materials

One and the same odor cue was used with the same participants in different experimental conditions and thus with different sets of English vocabulary. Thus, one and the same odor fragrance had to serve as cue for 60 (Class 1) or even 90 (Class 2) different words, which could theoretically have led to memory interference effects. Furthermore, former studies used one and the same odor cue for different items to learn.

Having this in mind, the efficacy of odor cues for consolidation and retrieval is surprising. It may be interesting to check whether it is possible to further increase odor cuing efficacy by using more odor cues.

### Timing of memory cues during sleep

Current theories about memory consolidation are based on numerous empirical findings^10–13,30^ and assume that memory consolidation implies a reactivation of hippocampal short-term memory traces and an integration into pre-existing cortical memory networks during slow wave sleep (SWS) at night^17,19,20,31^. Most studies indicate the necessity of controlling the sleep stages with EEG in order to be able to selectively apply the odor cues during the critical SWS stages. However, monitoring sleep stages and presenting cues only during SWS is a considerable operational expense with related difficulties for a potential practical application. Further, sleeping in the lab, wearing an EEG cap with many electrodes and cables associated, may influence sleep quality in a negative way. Our results are highly interesting in this context, as they show comparable memory benefits with continuous odor stimulation during the whole night. Furthermore, recent evidence indicates beneficial effects from whole-night odor cueing in a creativity task^32^. Thus, temporally selective cueing during sleep is apparently not a necessary precondition in order to achieve a beneficial effect of odor cueing at night, which is an important finding for practical application perspectives. However, we cannot rule out that a more specific cueing during certain sleep periods may have further increased the effectiveness of cueing.

Interestingly, memory cueing during night is not restricted to the olfactory modality, as auditory cues, even the auditory presentation of previously learned words during SWS, also work^13,19,20^. However, continuous stimulation with auditory cues can be problematic because of a certain refractoriness pattern: Auditory stimulus presentation needs to be below a certain temporal duration^33^ and spaced in time within the SWS stage in order to prevent the disappearance of the beneficial effect of cueing. The latter strongly reminds of well-known spacing effects^15,34–36^ or retroactive interference effects during learning^16^. Our continuous odor stimulation during night shows that the refractoriness problem is at least not that severe in the olfactory domain. Further auditory cueing may affect sleep quality and in the worst case wake the participants (e.g.^37^). So far, there is some evidence that sleep quality is not affected by odor cueing^38^. However, further studies are necessary to better clarify this important point.

## Conclusion

One desirable goal of memory research is to find an effortless way of selective learning during sleep. Studies on learning and memory have shown the crucial role of sleep during memory formation (e.g.^12,22,39^). Unfortunately, sleep does not replace learning effort. However, the series of experiments working with cues during memory encoding and during consolidation (TMR) indicate that we can at least optimize learning during sleep^13,19,20^. The present results provide further evidence that this is also possible in real life and with little effort. Moreover, our results indicate that odor cueing during the whole night and without sleep monitoring provides similar effect sizes as highly controlled lab studies; yet, the limited control of this field study leaves a number of questions unanswered. Further, although there is evidence that cueing during sleep does not affect sleep quality^38^, the question of potential side effects of cueing during sleep has not been sufficiently answered yet. We regard memory cueing during night together with its real-life applicability as highly promising for further basic research steps – perhaps with a higher degree of experimental control of field studies – and for easy application not only in educational contexts. Unfortunately, sleep alone is not sufficient enough and learning effort stays necessary. However, this line of research indicates that we can make our learning life easier while we sleep, and who would have thought that our nose can significantly help with this?

## Supplementary information

Supplementary Material.
