# Supplementary material for: How odor cues help to optimize learning during sleep in a real life-setting
Source: Sci Rep. 2020 Jan 27;10:1227. doi: 10.1038/s41598-020-57613-7 (PMC6985213; doi:10.1038/s41598-020-57613-7)
Supplement: Supplementary file 1 — Supplementary Material. [file 41598_2020_57613_MOESM1_ESM.pdf]

How odor cues help to optimize learning during sleep in a real life setting

Supplementary Materials

Franziska Neumann[1,2], Vitus Oberhauser[2], Jürgen Kornmeier[1,3,4]

[1]Institute for Frontier Areas of Psychology and Mental Health, Freiburg, Germany

[2]Faculty of Biology, University of Freiburg, Germany

[3]Department of Psychiatry and Psychotherapy, Medical Center, University of Freiburg

[4]Faculty of Medicine, University of Freiburg, Germany

Keywords: odor memory cueing, vocabulary learning, memory consolidation, slow-wave sleep, field research

Corresponding Author: Jürgen Kornmeier, juergen.kornmeier@uni-freiburg.de

Parametric tests like ANOVAs or t-tests are sensitive to outliers, potentially increasing alpha error probabilities. Visual inspection of Fig. 2 reveals some potential outlier candidates in the control group of condition LS (i.e. the top-most two blue circles), that may affect the statistical results of the present data (see dark green square in Supplementary Figure 1):

In order to get a rough estimate of how these data points contribute to the overall results as well as being motivated by the comments of one Reviewer, we calculated additional variants of statistics without the outliers and present the results in this Supplementary Information File.

Due to school internal organizational reasons, the LST condition was unfortunately not executed in Class 2. Consequently, only data from the N, LT and LS conditions were available for Class 2. For this reason we calculated two separate ANOVAs in the main manuscript.

ANOVA 1 contained the factor *condition* with the three factor steps N, LT and LS and was based on the data from Class 1 and Class 2. We re-calculated this ANOVA 1 without the two potential outliers mentioned above.

ANOVA 2 contained the factor *condition* with the two factor steps LS and LST and was based on the data from Class 1 only, since no data from Class 2 were available for condition LST. We also re-calculated this ANOVA 2 without the two potential outliers mentioned above.

Results ANOVA 1:

After removing the two outliers, the first ANOVA was re-calculated on conditions N, LT and LS with the data from both classes:

Overall, we found the same results with slightly smaller effect sizes: The ANOVA revealed no significant effect for the factor CLASS ($p = 0.144$, $F(1,48) = 2.21$, $\eta^2_p = 0.094$), nor for the factor GROUP ($p = 0.46$, $F(1,48) = 0.5$, $\eta^2_p = 0.026$) nor for condition ($p = 0.14$, $F(2,96) = 2.04$, $\eta^2_p = 0.04$). Without the data from these two participants, the interaction GROUP x CONDITION revealed a p-value of $p = 0.054$ ($F(2,96) = 3$, $\eta^2_p = 0.059$).

Results ANOVA 2 with conditions LS and LST:

We also re-calculated the second ANOVA without the two potential outliers from condtion LS. Since only data from one class were available for condition LS, this ANOVA was calculated on conditions LS and LST with the data from class 1 only.

The ANOVA revealed a significant effect for the factor GROUP ($p = 0.027$, $F(1,28) = 5.49$, $\eta^2_p = 0.39$), and for the factor CONDITION ($p = 0.02$, $F(1,28) = 5.79$, $\eta^2_p = 0.17$), but not for the interaction GROUP x CONDITION ($p = 0.14$, $F(1,28) = 2.3$, $\eta^2_p = 0.076$).

In summary, while the overall pattern of results stayed the same. After removing the two potential outliers, mentioned above, the two factors CONDITION and GROUP stayed significant, while the effect sizes decreased slightly. Interestingly, the p-value of the interaction decreased from $p = 0.44$ to $p = 0.14$.

Discussion

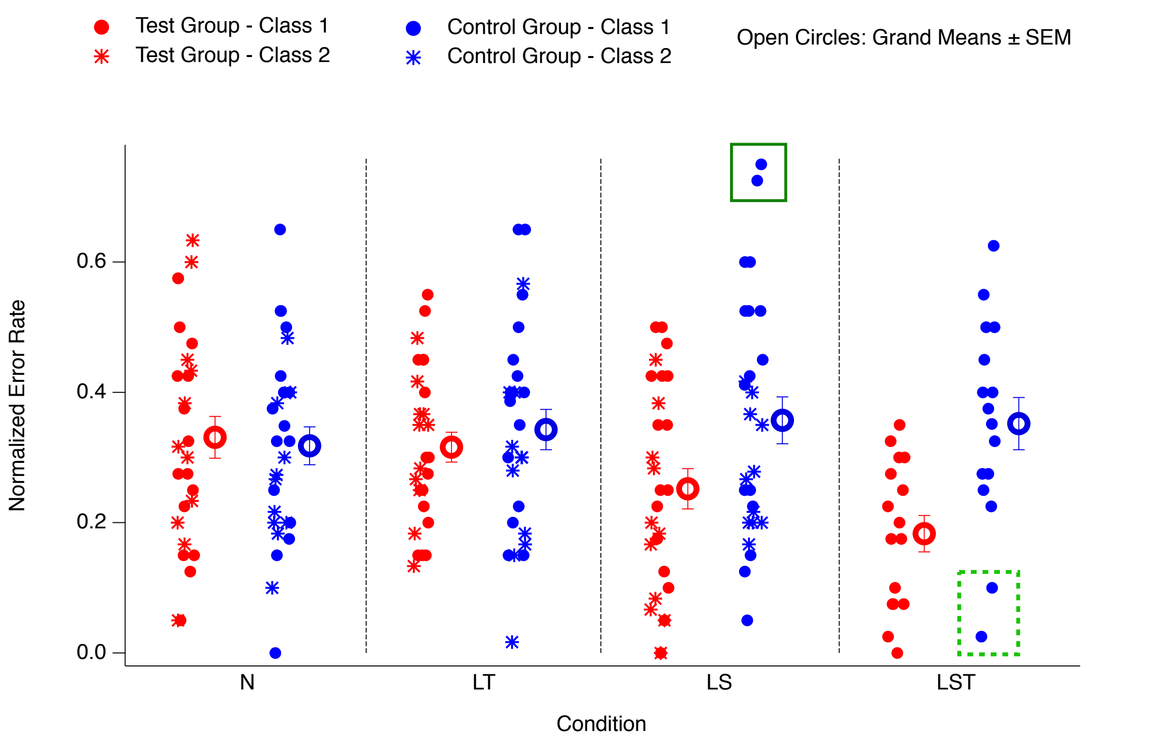

Supplementary Figure 1. Results: Small icons (stars and circles) represent data from individual participants. The data of the test group is in red, of the control group is blue. Larger open circles represent averages across classes ± SEM. Filled circles represent data from Class 1, stars represent data from Class 2. The different columns, separated by vertical dashed black lines, represent the different experimental conditions (N: no odor cue; LT: odor cue during learning and test; LS: odor cue during learning and during sleep; LST: odor cue during learning, sleep and test). Horizontal jitter across icons within monochrome sub-columns is due to presentation purposes. We found smaller numbers of errors LS and LST compared to conditions N and LT, and a tendency for smaller errors in LST compared to LS. Class 2 provided no data for condition LST. The ‘d’ indicates Cohen’s d as a measure of effect sizes. Data are normalized with respect to the number of vocabulary words tested in the respective vocabulary tests. Green rectangles indicate examples of potential outlier candidates.

Removing two potential outliers kept the overall pattern of results stable, while unsurprisingly decreasing effect sizes slightly. At a first glance, the question of whether additional memory cueing during the retrieval test (LST condition) had an additional effect on memory performance has to be responded negatively if this decision is based on the ANOVA result, because the ANOVA 2 revealed no CONDITION x GROUP interaction.

However, in our view it is worth to have a second view on these data and consider three potentially important points:

(1) p-values of the interaction decrease from 0.44 with the potential outliers to 0.14 without the potential outliers.

(2) Comparing post-hoc tests between groups revealed considerable effect size for the condition LST of Cohen's d = 1.22 and only about half this effect size for the comparison LS (d = 0.61).

(3) Statistics typically reflect signal-to-noise ratios. In our field study, an additional amount of noise may have been introduced by the fact that all the four conditions were based on different vocabulary material and thus on different final tests. This additional noise may have worsened the signal-to-noise ratio of the interaction between CONDITION and GROUP.

Identifying outliers is a critical issue, depending very often on arbitrary criteria and therewith on arbitrary decisions. This is particularly true for the current set of data. The two data points within the dark green square in the Supplementary Figure 1 may be interpreted as outliers. However, one could also argue about the data points in the light green dashed squares. One can calculate a number of different ANOVAs and post-hocs combining different outlier candidates and removing them. As a result, the overall picture becomes more and more complex, with the increasing number of different analysis variants. Further, removing outliers reduces the number of data points and can decrease the statistical power. It would have been beneficial to have data from Class 2 for the LST condition as well. However, the specific character of this field study made it unfortunately impossible to attach this condition belatedly as data for this condition hasn't been collected initially.

Given that defining outliers is always critical and that the choice of outliers is particularly not absolutely obvious in the current data, the more convincing strategy would be to replicate the present results in other labs. A replication in our lab is on the agenda. We hope that our manuscript may evoke additional replications in field studies from other groups.
